# *Cryptococcus laurentii* Diarrhea in a Neoplastic Patient

**DOI:** 10.1155/2015/216458

**Published:** 2015-01-27

**Authors:** Franco Calista, Federica Tomei, Pasquale Assalone, Divina Traficante, Gianni Di Pilla, Carla Pepe, Liberato Di Lullo

**Affiliations:** ^1^Medical Oncology Unit, ASReM, “F. Veneziale Hospital Isernia”, Via S. Ippolito No. 1, 86170 Isernia, Italy; ^2^Radiology Unit, ASReM, “F. Veneziale Hospital Isernia”, Via S. Ippolito No. 1, 86170 Isernia, Italy; ^3^Clinical Pathology Unit, ASReM, “F. Veneziale Hospital Isernia”, Via S. Ippolito No. 1, 86170 Isernia, Italy

## Abstract

We present a rare case of diarrhea and neutropenia caused by *Cryptococcus laurentii (C. laurentii)* infection in old patient with metastatic rectal cancer who underwent FOLFOX plus Cetuximab chemotherapy. *C. laurentii* is an extremely rare human pathogen. To the best of our knowledge, here, we report the first case of diarrhea and neutropenia caused by *C. laurentii* in a 74-year-old man with metastatic rectal cancer and hepatic metastases who underwent FOLFOX plus Cetuximab chemotherapy.

## 1. Introduction

*C. laurentii* is a rare human pathogen. This fungus was previously considered saprophytic and nonpathogenic to humans, but it has been isolated as the etiologic agent of skin infection, keratitis, endophthalmitis, lung abscess, peritonitis, meningitis, and fungemia [1, 2].

To the best of our knowledge the present report is the first to describe isolation of* C. laurentii* from human stool and this is the first reported isolation of* C. laurentii* in neoplastic patient's stool.

We present the case of a 74-year-old man who undergoes FOLFOX plus Cetuximab chemotherapy due to metastatic rectal cancer.

## 2. Case Report

We present the case of a 74-year-old Caucasian man with rectal cancer and hepatic metastases, severe carotid stenosis, and chronic hepatitis B. He smoked for forty years and quit twenty years ago.

In December 2013 the patient underwent colonoscopy and total body computed tomography scan. Histopathological and molecular examination showed a rectal adenocarcinoma with K-ras and N-ras wild type. In January 2014 FOLFOX plus Cetuximab chemotherapy was started.

**Figure 1:**
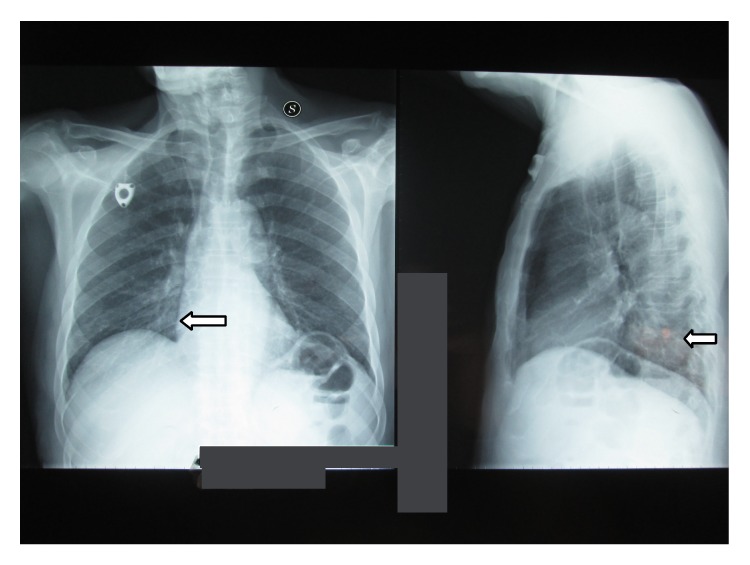
Chest X-ray at the admission.

**Figure 2:**
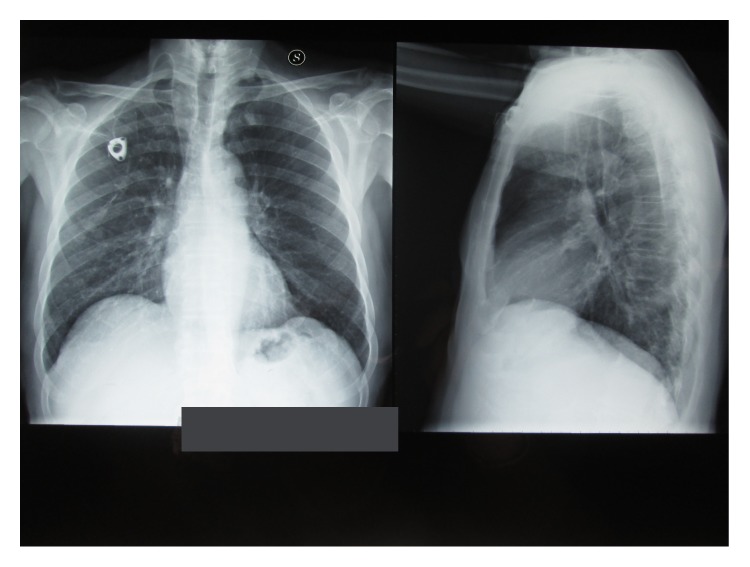
Chest X-ray after lipid formulation of amphotericin B therapy.

After the fifth cycle of chemotherapy the patient was admitted with nausea, vomit, and diarrhea. Systemic examination showed no fever or hypotension and dyspnoea. Blood test revealed an electrolyte imbalance. A chest X-ray showed right lower lobe pneumonia (Figure 1). Patient was empirically put on a third-generation cephalosporin plus ciprofloxacin; loperamide, rifaximin, and rehydration therapy were also initiated. On third day of admission laboratory investigations reported G1 leucopenia: white blood cell count: 3.310 K/*μ*L (reference range 4.0–10.0) and G2 neutropenia: neutrophil cell count: 1.240 K/*μ*L (reference range 2.5–7.5) and G2 anemia: haemoglobin: 8.0 g/dL (reference range 13.5–17.5).* C. laurentii* (+++) was isolated from stool culture on the seventh day of admission. At this point, lipid formulation of amphotericin B (AmBisome) 3 mg/Kg was started and continued for ten days. Anamnesis revealed that the patient had a goldfinch to which he attended every day. Echocardiogram, HIV test, and eye examination were performed with negative result. During treatment with lipid formulation of amphotericin B (AmBisome) we noticed a gradual improvement in leukoneutropenia up to the normalization of the values and disappearance of diarrhea. After ten days a stool culture for fungus and a chest X-ray were repeated with negative result (Figure 2). On the eighteenth day of admission the patient was discharged in good health. After one week the patient has continued FOLFOX plus Cetuximab chemotherapy without side effects.

## 3. Discussion

*C. laurentii* is a rare human pathogen. This fungus was previously considered saprophytic and not pathogenic to humans [1]. In the last few years there has been an increasing incidence of* C. laurentii* infection due to a growing population of immunosuppressed patients.* C laurentii*, a basidiomycetous encapsulated yeast, is present in the droppings and cloacal samples of feral birds, such as pigeons. Our patient had a goldfinch to which he attended every day.* C. laurentii* is responsible for both deep seated infections, such as fungemia and meningitis, and superficial infections such as keratitis [3]. Generally* C. laurentii* infection clinically presents with febrile illness, hypotension, cardiac valvular involvement, and choroidal infection and endophthalmitis [4, 5]. Our patient was admitted with diarrhea, and he had no fever or hypotension. Subramanian and Mathai [6] showed that the lung is most probably the initial portal of entry in immunocompromised patients. In our case, total body computed tomography performed before chemotherapy showed no pneumonia. After the isolation of* C. laurentii* in stool culture echocardiogram and eye examination were performed because cardiac valvular involvement, mycotic aneurysms, myocarditis, and endocarditis due to* C. laurentii* have been published. Our patient also underwent HIV test because Cryptococcosis is the first opportunistic infection that occurs in over a quarter of patients who develop AIDS [2, 6].

To the best of our knowledge the present report is the first to describe isolation of* C. laurentii* from human stool. Our patient was admitted with diarrhea and presented no chemotherapy-related neutropenia during admission.

## References

[1] Molina-Leyva A., Ruiz-Carrascosa J. C., Leyva-Garcia A., Husein-Elahmed H. (2013). Cutaneous *Cryptococcus laurentii* infection in an immunocompetent child. *International Journal of Infectious Diseases*.

[2] Banerjee P., Haider M., Trehan V. (2013). *Cryptococcus laurentii* Fungemia. *Indian Journal of Medical Microbiology*.

[3] Khawcharoenporn T., Apisarnthanarak A., Mundy L. M. (2007). Non-neoformans cryptococcal infections: a systematic review. *Infection*.

[4] Colmers R. A., Irniger W., Steinberg D. H. (1967). *Cryptococcus neoformans* endocarditis cured by amphotericin B. *The Journal of the American Medical Association*.

[5] Crump J. R. C., Elner S. G., Elner V. M., Kauffman C. A. (1992). Cryptococcal endophthalmitis: case report and review. *Clinical Infectious Diseases*.

[6] Subramanian S., Mathai D. (2005). Clinical manifestations and management of cryptococcal infection. *Journal of Postgraduate Medicine*.

